# Expression of a Synthetic Gene for the Major Cytotoxin (Cyt1Aa) of *Bacillus thuringiensis* subsp. *israelensis* in the Chloroplast of Wild-Type *Chlamydomonas*

**DOI:** 10.3390/biology7020029

**Published:** 2018-05-08

**Authors:** Seongjoon Kang, Obed W. Odom, Candice L. Malone, Saravanan Thangamani, David L. Herrin

**Affiliations:** 1 Pond Life Technologies LLC, Cedar Park, TX 78613, USA; joonk@utexas.edu; 2 Department of Molecular Biosciences, University of Texas at Austin, Austin, TX 78712, USA; owodom@utexas.edu (O.W.O.); candicemal@gmail.com (C.L.M.); 3 Department of Pathology, University of Texas Medical Branch, Galveston, TX 77555, USA; sathanga@utmb.edu

**Keywords:** biolarvacide, green alga, *Bacillus thuringiensis* subsp. *israelensis*, *Chlamydomonas*, Cyt1Aa, pest control, West Nile, Zika

## Abstract

*Chlamydomonas reinhardtii* (*Chlamydomonas*) strains that are toxic to mosquito larvae because they express chloroplast transgenes that are based on the mosquitocidal proteins of *Bacillus thuringiensis* subsp. *israelensis* (Bti) could be very useful in mosquito control. *Chlamydomonas* has several advantages for this approach, including genetic controls not generally available with industrial algae. The Bti toxin is produced by sporulating bacteria and has been used for mosquito control for >30 years without creating highly resistant mosquito populations. The suite of toxins is four main proteins: three Cry proteins and the cytotoxic Cyt1Aa (27 kDa). Cyt1Aa is not very toxic to mosquitoes by itself, but it prevents the development of resistance. The production of Cyt1Aa in other microbes, however, has been challenging due to its affinity for certain membrane phospholipids. Here we report on the production of recombinant Cyt1Aa (rCyt1A) in the chloroplast of photosynthetic *Chlamydomonas* at levels of at least 0.3% total protein. Live cell bioassays demonstrated toxicity of the rCyt1Aa *Chlamydomonas* to larvae of *Aedes aegypti*. We also expressed the chloroplast *cyt1Aa* gene in a wild-type *Chlamydomonas* strain (21 gr) that can grow on nitrate. These results have implications for developing a *Chlamydomonas* strain that will be toxic to mosquito larvae but will not induce strongly resistant populations.

## 1. Introduction

Mosquitoes are vectors for some important parasites and viruses that cause diseases in humans, which include the malarial parasite, plasmodium, and the emerging viruses, West Nile, chikungunya and Zika [1]. Since treatments for the aforementioned viruses are generally lacking, and notwithstanding the sexual transmission of Zika, vector control is essential for limiting the prevalence of these diseases. Mosquito control has proven to be an effective approach, that is both preventative and powerful because it blocks the transmission of multiple diseases simultaneously. The preferred strategy is integrated pest management (IPM), which targets all stages of the insect life cycle. The pesticides used in IPM are increasingly problematic, especially the chemicals that kill adult insects, which are toxic to non-target organisms, such as honey bees. Moreover, the development of chemically resistant mosquito populations is inevitable [2,3,4]. Thus, there is an urgent need for new, sustainable products for mosquito control.

All mosquitoes must develop in water, which is a much more restricted space than that occupied by flying adults. Moreover, killing larvae is more preventative than killing adults, which may have already transmitted disease by the time they are dispatched. Fortunately, a naturally occurring bacterium, *Bacillus thuringiensis* subsp. *israelensis* (Bti), produces a protein toxin during sporulation that specifically kills mosquito and black fly larvae. Moreover, this crystal-like protoxin has minimal non-target effects, and has not produced strong resistance in targeted mosquito populations in >30 years of use [5]. However, the Bti toxin has limitations, in that it does not persist very long in many environments, and in polluted or organic-rich waters its efficacy is diminished, requiring much higher amounts [6,7,8,9,10,11]. These two disadvantages stem from the fact that the Bti toxin is proteinaceous and extracellular, and thus can be inactivated by the environment. Applying the Bti spores is not efficacious as they do not reproduce well in most aquatic habitats. However, the elucidation of the Bti toxin at the molecular level has enabled an approach whereby the toxin could be delivered intracellularly (for protection) in a microbial host that grows in larval habitats [12,13,14,15,16]. Although such an organism has not yet been widely employed, there is still considerable interest in this approach [17,18,19].

Many microalgae are known to be important food sources for mosquito larvae [20,21,22], and a few cyanobacteria (*Agmenellum*, *Anabaena*, *Synechococcus*) and two eukaryotic green algae (*Chlamydomonas and Chlorella*) have been targeted for genetic modification to make them useful for larval control [12,13,14,15,16,17,18,19,23]. Our approach is to develop strains of *Chlamydomonas reinhardtii* (*Chlamydomonas*) that express genes in the chloroplast that are based on the Bti toxin. *Chlamydomonas* is an edible alga that is motile (so it can stay in the water column), grows in larval habitats, and is a genetic model system. Also, unlike cyanobacteria or most industrial eukaryotic algae, *Chlamydomonas* depends on sexual reproduction for continued survival in the environment; this is because the zygote, which is formed by fertilization of the two mating types, is the resilient stage of the life cycle [24]. Moreover, since sexual reproduction is accompanied by uniparental inheritance of the chloroplast [24], this feature can be exploited to prevent the formation of genetically modified progeny by engineering the chloroplast and using the minus mating type in the field. Taken together with the limits imposed by the environment on vegetative cells, e.g., their poor cold tolerance, there would be controls on both asexual and sexual reproduction of the modified algae. Finally, we note that chloroplast engineering in *Chlamydomonas* is well developed and can be accomplished without leaving behind bacterial antibiotic-resistance genes [25,26,27,28], which was a problem with earlier engineering projects [17].

The Bti toxin is composed of crystal-like inclusions containing mainly 4 proteins, Cry4Aa, Cry4Ba, Cry11Aa and Cyt1Aa. The larvae ingest the inclusion particles, and after dissolution and proteolytic processing at their termini, the activated proteins damage the gut membrane by inserting into the membranes and inducing ion leakage. Each of the proteins have some activity, but the Cry proteins are considerably more toxic than Cyt1Aa. However, Cyt1Aa is necessary to block the development of resistant populations. It achieves this by enabling the Cry proteins to bind to membranes in larvae that have become resistant through modification of Cry binding sites reviewed in [5].

Cyt1Aa is a pore-forming cytotoxin that binds to the phospholipids in gut membranes, principally phosphatidylcholine, sphingomyelin, and phosphatidylethanolamine [29]. Presumably for this reason, it has been difficult to express the *cyt1Aa* gene in heterologous systems, mostly because of toxicity to the host cells, but also because it can be unstable (in some systems) in the absence of a helper protein from Bti called P20 [13,30,31]. Since thylakoid membranes do not contain the aforementioned phospholipids [24], and chloroplasts have a variety of protein chaperones [32], we reasoned that there might be less of a problem expressing *cyt1Aa* in the chloroplast than in other systems. In our previous work, we expressed the Cry proteins in the chloroplast of *Chlamydomonas* using an inducible system, because preliminary results suggested that strong constitutive expression might be toxic to the host cell, and good results were obtained for Cry11Aa and Cry4Aa_700_ [19]. Although we initially engineered the *cyt1Aa* gene for inducible expression, we found that *cyt1Aa* could be expressed constitutively in the chloroplast (using a wild-type background) at substantial levels and with only a minor effect on growth rate. In addition, we demonstrate chloroplast transformation and *cyt1Aa* expression in a wild-type strain of *Chamydomonas* that can grow on nitrate as sole nitrogen source (21 gr).

## 2. Materials and Methods

### 2.1. Chlamydomonas Strains, Growth, and Chlorophyll Determination

The *C. reinhardtii* strains used for chloroplast transformation were from the *Chlamydomonas* Resource Center: CC-373 contains a deletion in the chloroplast *atpB* gene (ac-u-c-2-21) and is non-photosynthetic until transformed [33], whereas CC-1690 is R. Sager’s wild-type strain, 21 gr, which can utilize nitrate [24]. The standard medium for culture growth was Tris-acetate-phosphate (TAP) medium [24]. However, for the CC-373 transformations, minimal medium was used, which was made by adjusting the pH of TAP with HCl instead of HOAc. Cultures were grown in moderate continuous light at 24 °C. For liquid cultures, flasks that were ~40% full were used, and they were mixed continuously (~125 rpm). Cell counts were inferred from total chlorophyll measurements using the reference value of 4 mg total chlorophyll per 1 × 10^9^ cells, and total chlorophyll was measured as described previously [19].

### 2.2. DNA Constructs

The *cyt1Aa* DNA sequence of *B. thuringiensis israelensis* (NCBI NC_010076.1, nt 17372-18121, gene ID: 5759908) was optimized with the program Optimizer [34] and a codon-usage table for chloroplast genes of *Chlamydomonas reinhardtii*. Also, an 8-aa FLAG peptide (DYKDDDDK) was added to the C-terminus, and A297 was changed to T297 to remove the internal Nde I site. The gene was synthesized using 26 DNA primers that were 50 nt in length and overlapped flanking primers by 25 nt [35]. The DNA polymerase was Phusion (NEB, Ipswich, MA, USA), and the thermocycle program was 55 cycles of: 30 s at 94 °C, 30 s at 52 °C, and 30 s at 72 °C. The product was purified and used for PCR with outside primers to produce only the full-length gene; the thermocycle program was 23 cycles of 30 s at 94 °C, 30 s at 50 °C, and 30 s at 72 °C. The product was double-digested and cloned into pBluescript using the Nde I (5′) and Xba I (3′) sites. The *psbD_m_*:c*yt1Aa*:*psbA* gene was assembled in the low-copy vector pET-16B: the optimized *cyt1Aa* sequence was excised from pBluescript by cleaving with Xba I, blunting with Klenow DNA polymerase, and digesting with Nde I. It was ligated to pET-16B that had been cut with Bam HI, blunted with Klenow DNA polymerase, and digested with Nde I. The new plasmid, pET-Cyt1Aa, was confirmed by Sanger sequencing (University of Texas at Austin DNA Facility). For chloroplast expression, the *psbD_m_* (5′) and *psbA* (3′) regions used for expressing Cry genes [19] were ligated to *cyt1Aa* in pET-Cyt1Aa, creating the *psbD_m_*:c*yt1Aa*:*psbA* gene.

For transformation of strain CC-373, we modified a plasmid used previously for that purpose, P-655 [33]. P-655 was cut with Xho I and Xba I to remove the *rps7*:*aadA*:*rbcL* gene, and the ends were blunted with Klenow (plus dNTP’s), dephosphorylated, and ligated to the *psbD_m_*:c*yt1Aa*:*psbA* gene, which had been excised from pET-16B with Bam HI and blunted with Klenow. *E. coli* transformants were analyzed by restriction with Nde I to distinguish the orientation of the *psbD_m_*:c*yt1Aa*:*psbA* gene.

To transform the 21 gr wild-type strain (CC-1690), the *psbD_m_*:c*yt1Aa*:*psbA* gene was excised from pET-16B with Bam HI and cloned into the chloroplast expression vector, p322-483aadA. The p322-483aadA vector was created by inserting the recyclable selectable marker 483aadA [26] into plasmid P-322, which has a cpDNA fragment that extends from the Eco RI site in the 4th intron of *psbA* (Cr.psbA4) to the Xho I site in the Cr.LSU intron of the 23S rRNA gene (NCBI ref. NC_005353.1). The 483aadA marker has the *atpA*:*aadA*:*rbcL* gene that confers spectinomycin resistance flanked by a direct repeat (DR) of 483 bp. The insertion of the marker in p322 re-created the Bam HI site between the *psbA* and 5S genes, and this was used for inserting the Bam HI-excised *psbD_m_*:*Cyt1Aa*:*psbA* gene.

### 2.3. Chloroplast Transformation and DNA Analysis

Biolistic transformation using S550d gold carrier particles (Seashell Technology) was performed as described previously [19]. Selection was on TAP-minimal plates for strain CC-373 (because repair of the *atpB* deletion restores photosynthesis and growth on minimal medium), and on TAP + spectinomycin (300 µg mL^−1^) plates for transformation of wild-type strain CC-1690. For the CC-373 transformation, we obtained 20–100 transformants per plate, which equaled 40–200 transformants per shot, since each shooting was replated on two selective plates. Transformation of CC-1690 gave about half as many transformants as did CC-373. 

For analysis of the CC-373 transformants, DNA was extracted from cells grown on agar plates as described [36] and analyzed by PCR with *Taq* DNA polymerase (NEB) and the manufacturer’s buffer. The initial PCR test was with Cyt1Aa-internal primers, 886 (5′-ccaggcatatggaaaatttaaatcattgtccattagaagatattaaag-3′) and 899 (5′-tcatgtctagattatttgtcgtcgtcgtctttgtagtctaatg-3′), which bound to the 5′ and 3′ ends, respectively, of the Cyt1Aa coding sequence. Subsequent reactions used external primers that annealed adjacent to the gene-insertion site; these were 1009 (5′-cctcctaacggagcattaaaatc-3′), which bound to nt 159528–159550 of cpDNA, and 1008 (5′-ccaaattatatttgtcgtccacgag-3′), which bound to nt 160160–160136 of cpDNA. The external primers were used to test for homoplasmicity, as they gave a PCR product of 633 bp if there was no *psbD_m_*:c*yt1Aa*:*psbA* gene inserted and a product of ~1850 bp if it was present. As no antibiotic resistance marker was employed, 2 to 4 rounds of subcloning (selecting each time for the colonies with the most copies of the *psbD_m_*:c*yt1Aa*:*psbA* gene) was required for copy-correction between the 2 copies of the inverted repeat to produce homoplasmicity [33]. The subcloning was performed using regular TAP plates, rather than TAP-minimal, since homoplasmicity of the repaired *atpB* gene occurred quickly on the original TAP-minimal selection plate. Only about 10% or less of the transformants also became homoplasmic for the presence of the *psbD_m_*:c*yt1Aa*:*psbA* gene.

For analysis of the CC-1690 transformants, the DNA was also analyzed first by PCR with *cyt1Aa*-internal primers (886 and 899, described above), and then with external flanking primers to determine homoplasmicity. The flanking primers annealed upstream and downstream, respectively, of the co-inserted gene cassettes, *psbD_m_*:c*yt1Aa*:*psbA* and DR-*atpA*:*aadA*:*rbcL*-DR (where DR is a direct repeat of 483 bp) and were 863 (5′-taacccataaatagtttcaattggaa-3′, binding at position 147143–147168 of cpDNA) and 975 (5′-aatgcaaagtaccatcagatattgcta-3′, binding at position 147266–147241 of cpDNA), respectively. They gave a PCR product of 124 bp if there was no insertion (i.e., untransformed DNA) and ~4300 bp with the inserted genes. Selection for spectinomycin resistance by growth on TAP plates containing 300 µg mL^−1^ spectinomycin (in the light) hastened the attainment of homoplasmicity, and most of the transformants that survived this selection became homoplasmic.

### 2.4. Protein Extraction and Western Blotting

For the extraction of total cellular protein, 50 mL of cell culture was centrifuged at 900× *g* for 10 min (at RT), and the cells were resuspended in 1 mL of 100 mM Tris-HCl pH 8.5, 100 mM DTT, 7 mM benzamidine, and 5 mM EDTA pH 8.0. Then, 0.6 mL was removed and added to 0.4 mL of SDS solution (5% sodium dodecylsulfate, 30% sucrose, 0.025% bromophenol blue), and the lysates were stored at −70 °C. Total chlorophyll was determined from an aliquot of the cells and used to estimate protein using a mass ratio that we previously determined to be close to 1:10 chlorophyll:protein.

For SDS-PAGE, the solubilized cells, plus purified Cry11Aa [19] in some samples, were heated to 100 °C for 5 min before loading on 10% polyacrylamide SDS-PAGE gels along with a pre-stained protein ladder (PageRuler, Fermentas, Waltham, MA, USA) as size markers. After electrophoresis, the gels were soaked in cold transfer buffer (25 mM Tris base, 192 mM glycine, 5% methanol) for 15 min and then electrotransferred to a PVDF membrane (Hybond-P, GE HealthCare, Pittsburgh, PA, USA) using a Genie blotter (Idea Scientific, Minneapolis, MN, USA) at 12 V for 2 h at 4 °C [37]. The membrane was stained with Ponceau S to check the protein transfer, and then blocked overnight at 4 °C with 5% nonfat dried milk in TBS-T (20 mM Tris-HCl pH 7.6,150 mM NaCl plus 0.05% Tween 20). After washing twice with 0.5% nonfat milk in TBS-T for 5 min each, the membrane was incubated for 2 h (RT) with an anti-FLAG monoclonal antibody (M2) coupled to alkaline phosphatase (Sigma-Aldrich A9469, St. Louis, MO, USA) that was diluted 1:5000 in TBS-T plus 0.5% nonfat milk. After antibody incubation, the membrane was rinsed twice (2 min ea) with 50 mL of 0.5% nonfat milk in TBS-T, and then four times (10 min ea) with 50 mL of TBS-T. Finally, the membrane was rinsed with purified water for 5 min, overlaid with 2 mL of CDP-Star substrate (Invitrogen, Carlsbad, CA, USA), and incubated for 3 min. The substrate was decanted, the edges of the membrane blotted with a Kimwipe (Kimberly-Clark, Irving, TX, USA), and then put in a sheet protector to prevent drying. After 40 min, the membrane was exposed to X-ray film for periods ranging from 10 s to 3 min.

### 2.5. Mosquito Bioassay

The algal strains used for the live cell bioassay with mosquito larvae were grown in liquid TAP medium to 2–5 × 10^6^ cells/mL, except for the Cry11Aa strain which was grown in TAP-minus copper [19]. The cells were harvested by centrifugation, washed with 40 mL of once-distilled water, and resuspended in water to 1 × 10^7^ cells/mL. For each assay trial, 17 mL of the resuspended cells was added to 17 mL of once-distilled water containing 14–16 mosquito larvae, giving a total vol of 34 mL (with 5 × 10^6^ cells/mL of algae). The mosquitoes were 3rd instar larvae of *Aedes aegypti* (Galveston), which had been raised on mosquito diet (Carolina Biological) and then starved for 48 h prior to the assay. The plastic cups were covered with clear plastic wrap and secured with a rubber band to prevent the escape of adults. The assays were incubated at 27 °C under a 12 h:12 h light-dark cycle, and were monitored daily for 12 cycles, at which time all larvae had either died or developed into adults.

## 3. Results

### 3.1. cyt1Aa Gene Engineering and Chloroplast Transformation

The codon-optimized *cyt1Aa* gene had a codon adaptive index (compared to Chlamydomonas chloroplast genes) of 1, but the same amino acid sequence as Bti Cyt1Aa. An 8-amino acid FLAG tag was added to the C-terminus to enable detection with a commercial antibody; and it was expected to have little or no effect on toxicity as both ends of Cyt1Aa are cleaved away by larval gut proteases [38]. The expression signals from the *psbD* (promoter and 5′ UTR) and *psbA* (3′ UTR) genes were used so that the cyt1Aa gene could be expressed using the inducible system-which requires that the target gene have a *psbD* 5′ UTR and a special host strain [39]—or constitutively in a wild-type background. Since we obtained good expression in the constitutive system, we did not attempt the inducible system with this gene.

**Figure 1:**
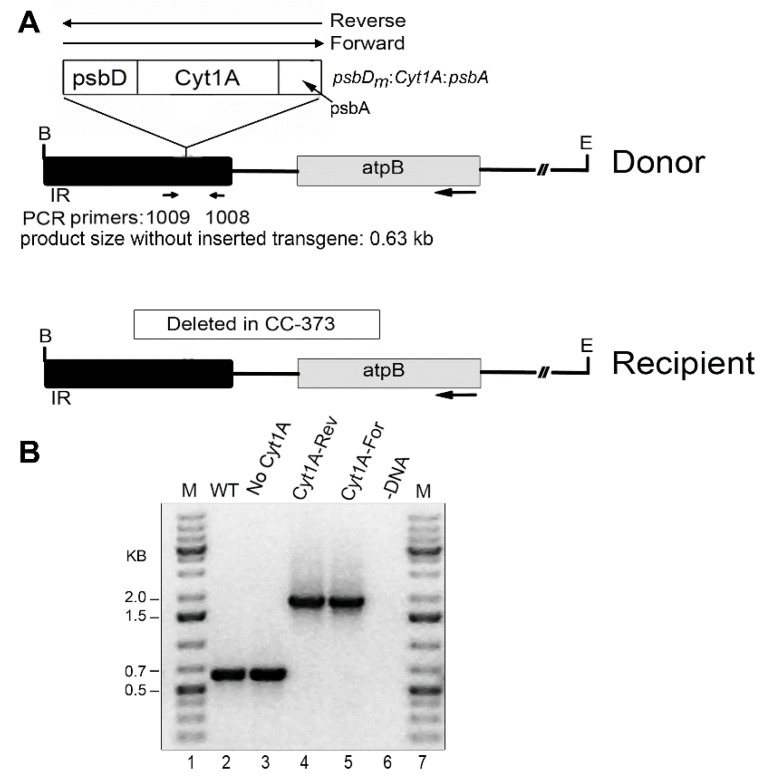
Introduced DNAs and analysis of homoplasmic *cyt1Aa*/CC-373 transformants by PCR. (**A**) Diagrams of the DNA constructs used for chloroplast transformation (Donor), and of the targeted region of the host, *Chlamydomonas* strain CC-373 (Recipient). In the Donor DNA, the *psbD_m_:cyt1Aa:psbA* gene was cloned into the IR region near *atpB* in both orientations, Reverse and Forward. The locations of the primers used for PCR (1009 and 1008) are indicated below the Donor DNA; these sites are created in Recipient DNA after repair of the deletion by homologous recombination with Donor DNA. B, E indicate restriction sites for Bam HI and Eco RI, respectively. The size of the PCR product if there is no transgene, 0.63 kb, is indicated; with the transgene, the expected product is ~1.8 kb (0.63 + 1.2). (**B**) PCR analysis of homoplasmic transformants. PCR was performed with total DNA from the indicated strains and primers 1009 and 1008. Lanes: M, DNA size markers; WT, CC-1690; No Cyt1A (CC-373 transformant that lost the *psbD_m_:cyt1Aa:psbA* gene by copy-correction with the other copy of the IR); Cyt1A-Rev (a transformant shot with Donor DNA that had *psbD_m_:cyt1Aa:psbA* in the Reverse orientation); Cyt1A-For (a transformant shot with Donor DNA that had *psbD_m_:cyt1Aa:psbA* in the Forward orientation); -DNA, minus-DNA control.

To achieve expression in a wild-type background, the *psbD_m_:cyt1Aa:psbA* gene was transformed into the chloroplast of the CC-373 strain, which has a deletion of the *atpB* gene [33]. This strain is non-photosynthetic due to the deletion, but the defect is complemented by transformation, thus producing a strain resembling wild-type, which can be selected on minimal medium. The transforming DNA simultaneously introduces the *psbD_m_:cyt1Aa:psbA* gene into the inverted repeat (IR) near the *atpB* gene (Figure 1). Since the *psbD_m_:cyt1Aa:psbA* transgene went into the cpDNA plasmid in both orientations relative to the orientation of the *atpB* gene (i.e., Reverse and Forward), we used both types of plasmids for transformation (see Donor DNA in Figure 1). Homoplasmicity is when all copies of the genome are the same, and this was achieved quickly at the atpB locus. However, several rounds of replating were required to achieve homoplasmicity at the *psbD_m_:cyt1Aa:psbA* locus, which because of the copy-correction process that occurs with the IR, can be completely retained, or lost in different clones. As shown in Figure 1, we obtained homoplasmic transformants for all 3 conditions, the reverse orientation of *psbD_m_:cyt1Aa:psbA* (Cyt1A-Rev), the forward orientation of *psbD_m_:cyt1Aa:psbA* (Cyt1A-For), and no *psbD_m_:cyt1Aa:psbA* gene (No Cyt1A). It should be noted that homoplasmicity for the integrated *psbD_m_:cyt1Aa:psbA* gene is indicated by the absence of the 0.63 kb PCR product, which contains the site of integration in the IR region.

### 3.2. Analysis of rCyt1Aa Protein Accumulation in the psbD_m_:cyt1Aa:psbA Transformants

**Figure 2:**
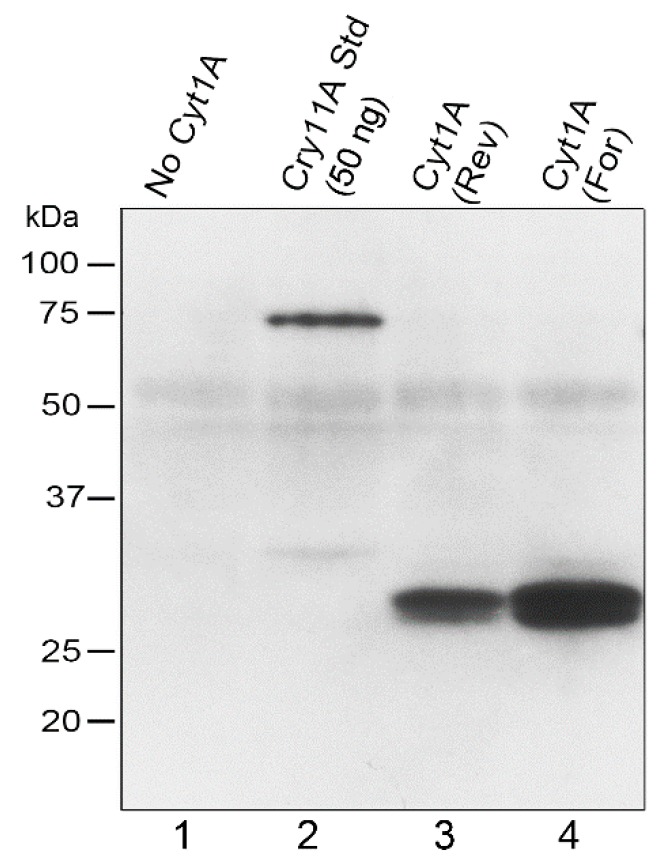
Western blot analysis of CC-373 transformants. 20 µg of total protein was loaded in each lane of the 10% polyacrylamide gel, which was electroblotted to PVDF and probed with an anti-FLAG antibody. The positions and sizes of protein markers are shown to the left. Lanes: No Cyt1A, total protein from a CC-373 transformant that did not have the *psbD_m_*:*cyt1Aa*:*psbA* gene; Cry11Aa Std (50 ng), same protein as in the “No Cyt1A” lane plus 50 ng of purified FLAG-tagged Cry11Aa as standard; Cyt1A (Rev), protein from a CC-373 transformant homoplasmic for the Reverse *psbD_m_*:*cyt1Aa*:*psbA* gene; Cyt1A (For), protein from a CC-373 transformant homoplasmic for the Forward *psbD_m_*:*cyt1Aa*:*psbA* gene.

Having obtained transformants that were essentially wild-type due to the complementation of the *atpB* gene, we examined the rCyt1Aa protein on western blots of total *Chlamydomonas* protein (Figure 2). Strong signals, relative to the standard (lane 2), and of the correct size, ~28 kDa, were obtained for both orientations of the *psbD_m_*:c*yt1Aa*:*psbA* gene, although the forward orientation produced more protein (Figure 2). The transformant that lacked the *psbD_m_*:c*yt1Aa*:*psbA* gene due to copy-correction with the other half of the IR during the cpDNA sorting-out period, gave no corresponding protein signal, as expected. The faint diffuse band of ~53 kDa in all the lanes is probably due to some non-specific sticking of the antibody to the very abundant proteins that migrate at that location; it serves as a fortuitous indicator of similar protein loading and transfer for the lanes. For lane 2, 50 ng of purified FLAG-tagged Cry11Aa [19] was added to the *Chlamydomonas* protein as a standard to provide a lower-limit estimate of FLAG-tagged Cyt1Aa. The results suggest that the *psbD_m_*:c*yt1Aa*:*psbA* transformants have at least 60 ng of rCyt1Aa per 20 µg total protein, or at least 0.3% of total protein is rCyt1Aa.

### 3.3. Growth Rates for the psbD_m_:cyt1Aa:psbA Transformants

**Figure 3:**
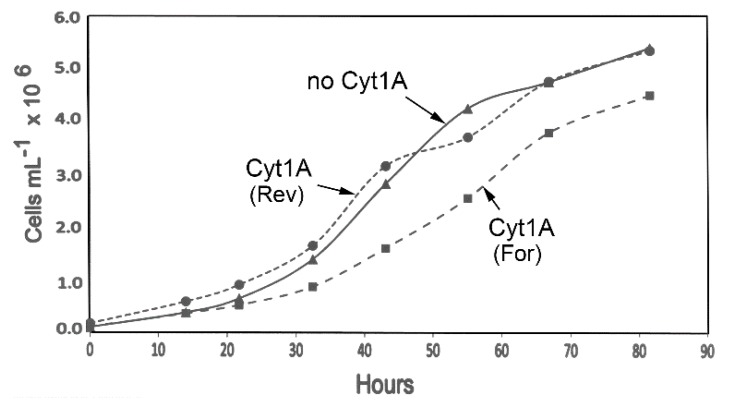
Growth curves for the *cyt1Aa*/CC-373 transformants. The indicated homoplasmic transformants were grown in 100-mL TAP cultures in 250-mL flasks as described in Methods. At the indicated times, aliquots (1 mL) were withdrawn, and total chlorophyll was quantified and converted to cell density.

Although it was apparent that the *psbD_m_*:*cyt1Aa*:*psbA* transformants grew well, it was possible that the *psbD_m_*:*cyt1Aa*:*psbA* gene (or rCyt1Aa protein) could be having a negative effect on the cells, and that it might show up in growth rate analysis. Therefore, liquid cultures of the same transformants analyzed in Figure 1 and Figure 2 were monitored for ~82 h during growth in the light in standard TAP medium (Figure 3). As the data shows, the growth curve for the *psbD_m_*:*cyt1Aa*:*psbA* transformant in reverse orientation, Cyt1A (Rev), is very similar to the control strain lacking the *cyt1Aa* gene (no Cyt1A). However, the *psbD_m_*:*cyt1Aa*:*psbA* transformant in the forward orientation, Cyt1A (For), which had the higher level of Cyt1Aa protein (Figure 2), grew at a rate that was ~20% slower. The doubling time was ~10 h for the control (no Cyt1A) and Cyt1A (Rev) strains, and ~12 h for the Cyt1A (For) strain. This result suggests that there is a lack of rCyt1Aa toxicity at the level found in Cyt1A (Rev), but there is some host toxicity with the higher level of rCyt1Aa found in the Cyt1A (For) transformant.

### 3.4. Bioassay with Aedes Aegypti Larvae

Although Cyt1Aa has relatively poor toxicity to mosquito larvae by itself [5,13,31], it has been reported to be detrimental and even lethal in several cases [5]. To assess our rCyt1Aa-producing algae, we performed larval bioassays where 14–16 3rd instar larvae of *Aedes aegypti* (*Galveston*) were incubated with live algal cells in dH_2_0. Under these conditions, the algae do not grow even though they are incubated under a 12 h:12 h light-dark cycle at 27 °C; moreover, they are readily consumed by hungry larvae. The initial assays ran for up to 72 h, and we saw a small increase in larval mortality with the rCyt1Aa strains (not shown). However, with a longer time frame, ~1/3 of the larvae died (Table 1) with the rCyt1Aa strains, versus 1/8th for the No Cyt1A control. For a positive control, we used a highly toxic Cry11Aa strain created previously that produces Cry11Aa under inducing conditions to ~0.3% of total protein (is rCry11Aa) [19]; that strain was again highly toxic, killing ~88% of the larvae.

### 3.5. Transforming a Wild-Type (WT) Strain Capable of Growth on Nitrate

Nearly all published papers that involve transforming the chloroplast in *Chlamydomonas* use host strains that are derivatives of the 137c or Ebersold/Levine line of *C. reinhardtii*, which is incapable of growing on nitrate as sole nitrogen source because it lacks nitrate reductase due to mutations [24]. Since the ability to grow on nitrate could be important for a *Chlamydomonas* strain that was to be used for mosquito control, we have explored transforming the chloroplast of CC-1690, better known as Ruth Sager’s wild-type strain, or 21 gr. This strain is wild-type for nitrogen assimilation and can grow on nitrate [24].

**Figure 4:**
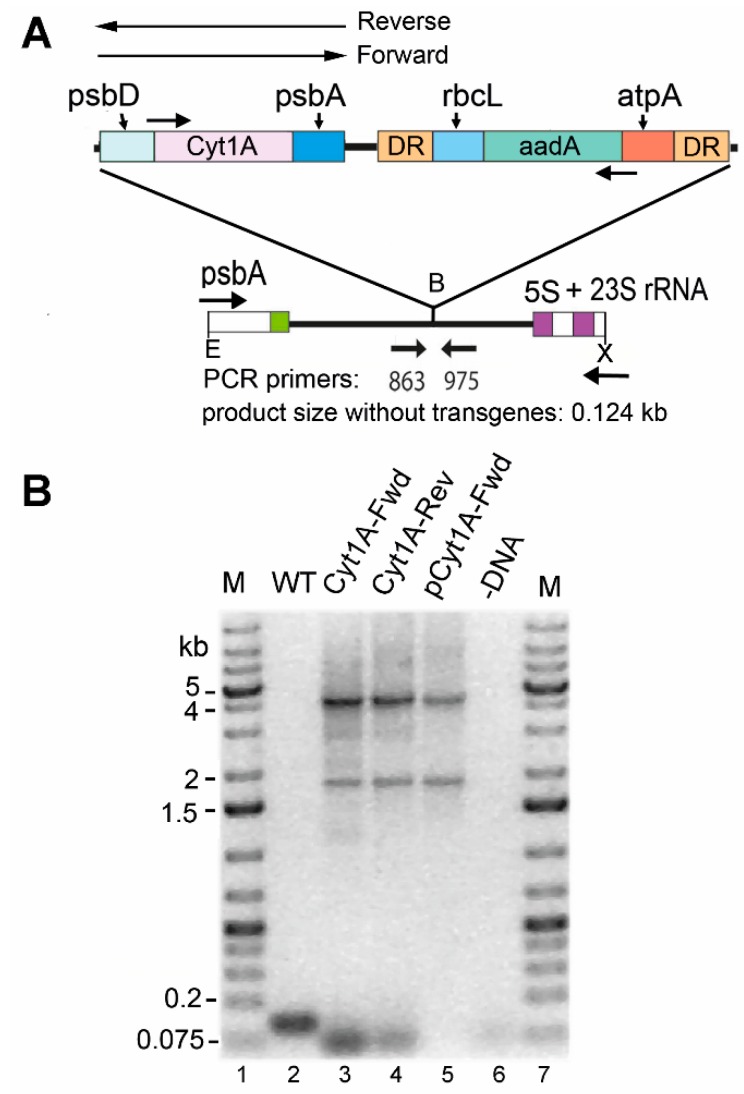
Introduced DNAs and analysis of homoplasmic *cyt1Aa*/CC-1690 transformants. (**A**) Diagram of the DNAs transformed into the chloroplast and the targeted region in the host, CC-1690 (WT). The arrows above the map indicate that the *psbD_m_*:*cyt1Aa*:*psbA* gene was cloned separately in both orientations, Reverse and Forward. The selectable marker gene, *atpA*:*aadA*:*rbcL*, which was only in the Reverse orientation, is flanked by 483-bp direct repeats (DR). E and X are restriction sites Eco RI and Xho I, respectively. (**B**) PCR analysis of homoplasmic transformants. PCR was performed with total DNA from the indicated strains (and plasmid) with primers (863 + 975) that flank the inserted DNA, to test for homoplasmicity. Lanes: M, DNA size markers; WT, CC-1690 host strain; Cyt1A-Fwd, transformant with *psbD_m_*:*cyt1Aa*:*psbA* in the Forward orientation; Cyt1A-Rev, transformant with *psbD_m_*:*cyt1Aa*:*psbA* in the Reverse orientation; pCyt1A-Fwd, plasmid that was transformed into CC-1690 to create the Cyt1A-Fwd transformant; -DNA, control reaction minus-DNA. Note: The ~1.8 kb band in lanes 3–5 is most likely a PCR artifact mediated by the large direct repeat (DR) at the ends of the selectable marker, which causes a deletion of the sequence between the DRs plus one copy of the DR, or ~2.5 kb.

To transform the chloroplast of CC-1690 (21 gr), we used an antibiotic resistance marker (483aadA) that also has the potential to be removed later by growth under non-selective conditions [26]. We elected to introduce the *psbD_m_*:*cyt1Aa*:*psbA* gene, since it worked well in CC-373 above, and in both orientations relative to the *aadA*-based marker, which confers spectinomycin resistance. The genes were integrated at a different location than in CC-373 (Figure 1), between the *psbA* and 5S rRNA genes in the IR (Figure 4A), but homoplasmic transformants were identified similarly, by amplifying the integration site using flanking primers (Figure 4B). The small (~0.124 kb) product that would be expected with these primers if there was no integration is completely absent in the Cyt1A-Fwd and Cyt1A-Rev transformants, because they are homoplasmic for integration. The 4 kb product is the size expected for amplification of both genes in tandem, whereas the ~1.9 kb product is an artifact of PCR, caused by the sizable (483 bp) direct repeat (DR) at the ends of the *atpA*:*aadA*:*rbcL* marker. Consistent with this notion is the fact that this product was also obtained with the plasmids that were introduced into the cells, and the result with pCyt1A-Fwd is shown (Figure 4B, lane 5). In summary, homoplasmic transformants of CC-1690 with the *psbD_m_*:*cyt1Aa*:*psbA* gene were obtained for further analysis.

### 3.6. Expression and Growth Characteristics of the CC-1690 Transformant

**Figure 5:**
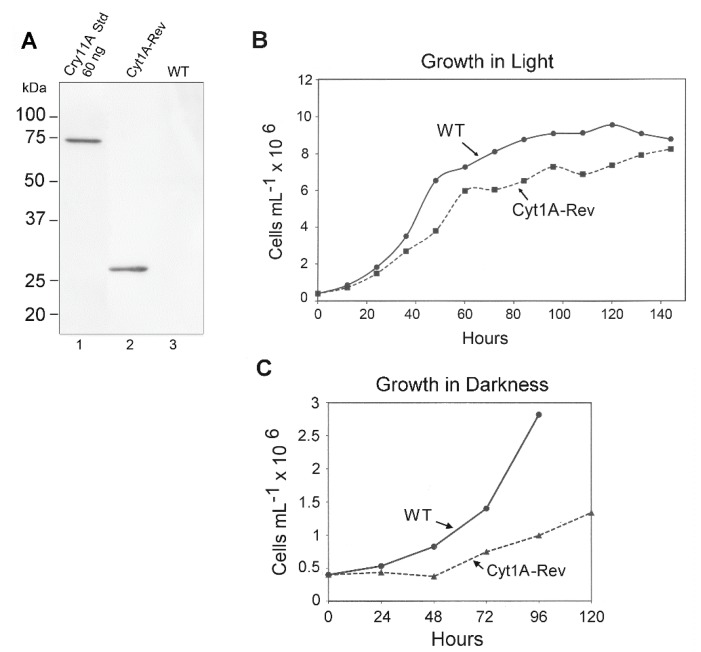
Western blot and growth rate analysis of the Cyt1Aa-Rev/CC-1690 transformant. (**A**) Western blot analysis of the Cyt1A-Rev transformant and the control WT strain (CC-1690); lane 1 contained ~60 ng of the FLAG-tagged Cry11Aa standard protein. The lanes were loaded with 20 µg of *Chlamydomonas* protein, including lane 1, which received 20 µg of WT protein. (**B**) Growth curves for the Cyt1A-Rev transformant (in CC-1690) and WT strain (CC-1690) in continuous light. The cells were grown in TAP medium in continuous light as described in Methods. At the indicated times, aliquots (1 mL) were withdrawn, and total chlorophyll was quantified and converted to cell density. (**C**) Growth curves for the Cyt1A-Rev transformant (in CC-1690) and WT strain (CC-1690) in continuous darkness. The cells were grown in TAP medium as described in Methods, except in continuous darkness. Total chlorophyll was quantified on aliquots removed at the indicated times and converted to cell density.

Western blotting was used to gauge the expression of the *psbD_m_*:*cyt1Aa*:*psbA* gene in CC-1690 using the FLAG antibody and total *Chlamydomonas* protein. Since both orientations of the *psbD_m_*:*cyt1Aa*:*psbA* gene gave levels of rCyt1Aa protein on western blots that were indistinguishable (not shown), only one orientation was analyzed for Figure 5. A single major band of the expected size, ~28 kDa, was obtained, and comparing it to the FLAG-tagged standard (60 ng of Cry11A Std, lane 1) the level of rCyt1Aa appears to be about 60 ng per 20 µg total protein, or ~0.3% of total protein.

We also examined the growth rate of the rCyt1Aa strain compared to the parental WT strain (CC-1690) in TAP medium under continuous light. The initial analysis was performed over about 80 h and growth of the rCyt1Aa strain was slower than WT (not shown). However, to see if the Cyt1Aa strain would eventually make it to a final density similar to WT, we monitored another set of cultures for 150 h (6.25 days) (Figure 5B). The rate of growth of the rCyt1Aa strain was again ~20% slower than WT, but it eventually reached a cell density (8 × 10^6^ cells/mL) close to that of WT (8.2 × 10^6^ cells/mL).

The reduced growth rate of the *cyt1Aa* transformant of CC-1690 (Figure 5B) and of the *cyt1Aa* transformant of CC-373 (Cyt1A (For) in Figure 3) suggests that there is some weak toxicity associated with producing rCyt1Aa in the chloroplast at substantial levels. However, there was at least one other explanation, that the *psbD_m_*:*cyt1Aa*:*psbA* mRNA was competing with the *psbD* mRNA for the translational factors that bind to the *psbD* 5′ UTR [40] and reducing *psbD* expression. As a simple test of this hypothesis, we examined growth curves of the *cyt1Aa* transformant (in CC-1690) and WT in the dark, where *psbD* expression levels should matter very little, because there is no photosynthesis. As Figure 5C shows, the growth rate of Cyt1Aa was still slower than WT, and possibly even more pronounced. This suggests that the reduction in growth rate of the *cyt1Aa* transformant compared to wild-type is probably due to some toxicity associated with producing Cyt1Aa in the chloroplast at substantial (>0.2%) levels.

## 4. Discussion

We have demonstrated that the cytotoxic Cyt1Aa protein from the mosquitocidal bacterium, Bti, can be produced in the chloroplast at reasonably high levels in wild-type photosynthetic cells. Although the CC-373 host strain was not photosynthetic before chloroplast transformation, it was after introducing the *psbD_m_*:*cyt1Aa*:*psbA* and *atpB* genes, the latter of which repaired the deletion in CC-373 and restored photosynthesis [33]. Although we probably could have achieved a higher level of rCyt1Aa with the inducible system [19,39]—because with that system the *psbD_m_*:*cyt1Aa*:*psbA m*RNA would not compete with *psbD* mRNA for translation factors that bind the *psbD* 5′ UTR [40]—we would still have had to develop conditions for *cyt1Aa* expression in a wild-type background. Inducible expression of toxin would not be desirable in a mosquito-control strain, since induction conditions would probably not be present in diverse field sites. Thus, these results have direct implications for developing a larvacidal *Chlamydomonas* strain that will not engender strong resistance because it produces Cyt1Aa constitutively [5].

We also expressed the *psbD_m_*:*cyt1Aa*:*psbA* gene in the chloroplast of a wild-type strain (21 gr, CC-1690) that can grow on nitrate as sole nitrogen source. To our knowledge, this is one of a very few, if not the only, report of chloroplast transformation that does not involve a 137c (Ebersold/Levine) line. *Chlamydomonas* strains carried in captivity can change due to genetic drift, and the 137c line lost nitrate reductase, which is needed for nitrate assimilation, prior to it becoming the favored strain for chloroplast researchers [24]. Although *Chlamydomonas* can grow on ammonia or even certain amino acids as nitrogen sources, these compounds can be limiting for growth in many natural habitats. Thus, a strain with great versatility in nitrogen utilization, such as 21 gr, should be better at growing in diverse larval habitats.

We were fortunate that the *psbD_m_*:cy*t1Aa*:*psbA* gene expressed well in a wild-type strain, as high-level expression of transgenes in wild-type *Chlamydomonas* chloroplasts remains a challenge. Indeed, the Cry11Aa and Cry4A_700_ genes that expressed well in the inducible system [19], expressed poorly when they were transformed into wild-type strains (S. Kang, O.W. Odom and D.L. Herrin, unpublished results). Why the *psbD_m_*:*cyt1Aa*:*psbA* gene expresses well in the presence of a normal *psbD* mRNA, while the *psbD_m_:cry11A:psbA* and *psbD_m_:cry4A_700_:psbA* genes do not is not clear, but it indicates that the coding region can be critical in transgene expression. Moreover, the answer is not as simple as a more-compatible coding region (with the *psbD_m_*:*cyt1Aa*:*psbA* gene), because expression of *cyt1A* using two other promoter-5′ UTR combinations (*atpA* and *16S*-*atpA*, respectively) did not yield high levels of rCyt1Aa in the CC-373 background (O.W. Odom and D.L. Herrin, unpublished results). Perhaps there are interactions between the 5′ UTR and coding regions of certain chloroplast mRNAs that affect their levels and/or translational efficiency.

Toxicity to heterologous hosts, and in some cases instability, has hindered transgenic *cyt1Aa* expression; however, in bacteria this was overcome by co-expressing another protein from Bti called P20 [13,31], which may be a chaperonin-like protein [32]. Since the chloroplast generally lacks the membrane phospholipids that attract Cyt1Aa, we posited that host toxicity might be less of a problem with chloroplast expression, and that seems to be the case, as we readily obtained chloroplast transformants producing rCyt1Aa. Moreover, we have not seen evidence of genetic instability with these transformants.

However, in two of the three Cyt1Aa transformants that we examined closely, there was evidence of a reduced growth rate (10–20%) in liquid culture relative to the control strain. Although that would not be a desirable trait for a strain that was to be used for mosquito control, we are not overly concerned at this point, because we are looking to co-express *cyt1Aa* with either *cry11Aa* or *cry4Aa_700_*, which are known to interact with Cyt1Aa [5] and could mitigate damaging effects of rCyt1Aa on the chloroplast. If there is still evidence of rCyt1Aa toxicity in the co-expressing strains, we can localize rCyt1Aa within the organelle, to keep it away from membranes, and that may prevent such damage. To that end, we have explored binding rCyt1Aa to starch granules using a starch-binding domain [41]. In preliminary experiments, the starch-binding domain eliminated the growth reduction in the CC-1690 strain caused by *cyt1Aa* expression, but it also reduced the level of the Cyt1Aa fusion protein, making it difficult to determine what actually restored the growth rate (S. Kang, O.W. Odom and D.L. Herrin, unpublished results). Nonetheless, this remains a potential approach to deal with Cyt1Aa host toxicity.

## 5. Conclusions

We have shown that a synthetic *cyt1Aa* gene can be expressed constitutively in the chloroplast of an essentially wild-type *Chlamydomonas*, with the rCyt1A protein (28 kD) accumulating to levels of at least 0.3% total protein. The rCyt1Aa strain was also shown to be toxic to larvae of *Aedes aegypti*, although not nearly as toxic as a Cry11Aa-producing strain, which was expected. We also expressed the *cyt1Aa* gene in the chloroplast of a wild-type strain that can grow on nitrate as sole nitrogen source (CC-1690 or 21 gr), which is a trait that could be important for a strain that is to be used for larval control in aquatic habitats. To that end, we are currently working to produce strains that constitutively express genes for both Cry11Aa and Cyt1Aa (at high levels) in the same chloroplast, and in a wild-type strain such as CC-1690.

## Figures and Tables

**Table 1:** Summary of bioassay with *Aedes aegypti* larvae fed live *Chlamydomonas*.

Chlamydomonas Strain ^a^	Larval Mortality	Pupae Formed	Adults Formed
No Cyt1A	2/16	14/16	14/16
Cyt1A (Rev)	7/16	9/16	9/16
Cyt1A (Fwd)	5/16	11/16	9/16
Cry11Aa induced	12/14	2/14	2/14

^a^ The algal cells were at 5 × 10^6^ cells/mL, plus 14 or 16 larvae. The Cry11Aa strain [19] was grown without copper to induce the *psbD_m_*:c*ry11A*:*psbA* gene.
